# Ameliorative Effect of Surface Proteins of Probiotic Lactobacilli in Colitis Mouse Models

**DOI:** 10.3389/fmicb.2021.679773

**Published:** 2021-09-03

**Authors:** P. R. Chandhni, Diwas Pradhan, Kandukuri Sowmya, Sunny Gupta, Saurabh Kadyan, Ritu Choudhary, Archita Gupta, Ganga Gulati, Rashmi Hogarehalli Mallappa, Jai K. Kaushik, Sunita Grover

**Affiliations:** ^1^Molecular Biology Unit, Dairy Microbiology Division, ICAR-National Dairy Research Institute, Karnal, India; ^2^Animal Biotechnology Center, ICAR-National Dairy Research Institute, Karnal, India

**Keywords:** surface proteins, colitis mice, anti-inflammatory, probiotics, lactobacilli

## Abstract

The increase in concern from viable cells of probiotics specifically in acute inflammatory conditions has led to the emergence of the concept of postbiotics as a safer alternative therapy in the field of health and wellness. The aim of the present study was to evaluate the efficacy of surface proteins from three probiotic strains in dextran sodium sulfate and trinitrobenzenesulphonic acid = induced colitis mouse models. The molecular weight of total surface proteins extracted from the three probiotic strains ranged from ∼25 to ∼250 kDa with the presence of negligible levels of endotoxins. Surface layer proteins (SLPs) (∼45 kDa) were found to be present only in the *Lactobacillus acidophilus* NCFM strain. In the *in vivo* study, significant differences were not observed in the weight loss and general appetite, however, the decrease in colon length was apparent in TNBS colitis control mice. Further, the administration of these surface proteins significantly reversed the histopathological damages induced by the colitogens and improved the overall histological score. The oral ingestion of these surface proteins also led to a decrease in myeloperoxidase activity and TNF-α expression while the IL-10 levels significantly increased for the strain NCFM followed by MTCC 5690 and MTCC 5689. Overall, the present study signifies the ameliorative role of probiotic surface proteins in colitis mice, thereby, offering a potential and safer alternative for the management of inflammatory bowel disorders.

## Introduction

With the growing concern in self-care and optimum health at all ages, recognition of a link between probiotics and health has become stronger. Probiotics confer health benefits primarily by improving the gut immune system, gut barrier integrity, competitive adhesion to the Gastro-intestinal mucosa, pathogen exclusion, secretion of host antimicrobial components such as β-defensins, and finally establishing the homeostasis in the gut ecosystem (Azad et al., 2018). Several studies have delved into the aspect of altering gut microbiota with probiotics as an approach to prevent or treat such diseases such as cardiovascular diseases, obesity, diabetes, etc (Thushara et al., 2016). Even though they are regarded as safe for use in human, food, and animal feed applications, there is a growing concern regarding their safety particularly under clinical conditions. Several clinical reports have revealed their involvement in opportunistic infections, infective endocarditis, sepsis, bacteremia, antibiotic resistance gene transfer, abdominal abscesses, meningitis, urological infections, and rheumatic vascular diseases, especially in patients receiving antibiotic treatment or those who are immunocompromised (Pradhan et al., 2020). Besides, the functionality is being affected irrespective of their viability and this could imply that the control of cell viability is not always enough to guarantee the functionality of a strain (Vinderola et al., 2011).

To circumvent the above challenges, the alternative safer option of using non-viable postbiotic components derived from specific probiotic strains that can improve the functionality of the gut system just as the live probiotic cells holds great promise. Most of these postbiotic components include heat killed cells of probiotic bacteria, cell wall components like wall teichoic acid (WTA), Lipoteichoic acid (LTA), and Peptidoglycan (PG), including probiotic cells containing metabolites like short chain fatty acids (SCFA), bacteriocins, vitamins, bioactive peptides, cell surface proteins like surface layer proteins (SLPs), mucus binding protein (MUB), fibronectin binding protein (FnBP), mannose specific binding protein, etc (Kim et al., 2017). The cell surface proteins extracted from probiotic bacteria are one of the promising groups of postbiotic components comprising of SLPs, MUB, FnBP, mannose specific binding protein, collagen binding protein, etc. The effects of cell surface proteins are incredibly diverse and have been shown to play a key role in many of the probiotic functionality such as adhesion to the host cells, strengthening of the gut barrier integrity, pathogen exclusion, stimulation of the host mucosal system to improve mucus production, and secretion of defense molecules such as β defensins (Gerbino et al., 2015; Singh et al., 2018). Furthermore, it has been demonstrated that the surface proteins, particularly SLPs of probiotic lactobacilli, exert protective immune regulation *via* regulatory signals in the intestinal epithelial cells that result in mitigation of colitis (Lightfoot et al., 2015).

The test probiotic strains, namely Lactoplantibacillus plantarum MTCC 5690 (previously Lactobacillus plantarum MTCC 5690 and Lactobacillus plantarum Lp91), Limosilactobacillus fermentum MTCC 5689 (previously Limosilactobacillus fermentum MTCC 5689 and Lactobacillus fermentum Lf1) and Lactobacillus acidophilus NCFM have been well-established for their immunomodulatory and anti-oxidative properties in the treatment of chemically [dextran sodium sulfate (DSS) and trinitrobenzenesulfonic acid (TNBS)] induced experimental colitis models (Sudhakaran et al., 2013; Chauhan et al., 2014; Pradhan et al., 2019b) besides its safety properties (Pradhan et al., 2019a). Hence, it could be postulated that the cell surface proteins of these probiotic bacteria may also hold immense potential for possible applications as biotherapeutic agents against inflammatory disorders such as colitis, Crohn’s disease, pouchitis, etc. Hence, the present study was designed to investigate the ameliorative effects of total surface proteins derived from the three probiotic strains viz. Lpb. plantarum MTCC 5690, Lm. fermentum MTCC 5689 and the reference probiotic strain L. acidophilus NCFM in both DSS and TNBS colitis mouse models.

## Materials and Methods

### Bacterial Strains and Culture Conditions

The two test probiotic strains *Lpb. plantarum* MTCC 5690 and *Lm. fermentum* MTCC 5689, and the standard probiotic strain *L. acidophilus* NCFM were procured from the repository of probiotic cultures, Molecular Biology Unit, ICAR-National Dairy Research Institute, Karnal, Haryana and maintained for routine use as streak plates in MRS agar and also preserved as glycerol stocks (20%) at −80∘C. The purity of the test probiotic strains was always checked microscopically by Gram’s staining, negative staining, and catalase test following standard protocols.

### Extraction and Quantification of Total Surface Proteins From Probiotic Strains

The total surface proteins from the three probiotic strains were extracted by Lithium Chloride (LiCl) method (Turner et al., 1997). The probiotic strains were initially activated in 10 ml MRS broth (37°C for 24 h), followed by sub culturing in 100 ml MRS broth and further scaling up to 500 ml MRS broth. The active cultures were pelleted out by centrifuging at 7,500 rpm for 20 min. The pellet so obtained was dissolved in 5 ml of 5 M LiCl (Sigma-Aldrich Co., United States) and further kept under refrigerated incubation with intermittent mixing for 2 h, after which the culture was centrifuged at 4,500 rpm for 15 min to extract out the surface proteins. The supernatant so obtained was carefully transferred to a 10 kDa cut off dialysis membrane (Sigma Chem Co., United States) and was allowed to dialyze against distilled water containing isopropanol and 100 mM [phenyl methyl sulphonyl fluoride (PMSF), protease inhibitor] for 48 h at 4°C. The distilled water was replaced once after 24 h. The dialyzed protein extract was then concentrated in a freeze drier and preserved under −20°C. The surface proteins extracted were quantified using the protocol of Lowry et al. (1951).

### Characterization of Extracted Surface Proteins From Probiotic Lactobacilli

#### Protein Profiling by SDS-PAGE

The extracted total surface proteins from the probiotic strains were characterized for its size and type using SDS-PAGE (12% gel) protein profiling following standard protocol (Lightfoot et al., 2015). The gel imaging was done in 2D gel documentation system (Epson Scanner; Proteomics images and spot picker, M/S GE Health care bioscience Pvt., Ltd., New Delhi).

#### Identification of Surface Proteins by Mass Spectrometry

The protein bands of around 45–50 kDa obtained from the probiotic strains (*Lpb. plantarum* and *Lm. fermentum*) were excised from the gel and samples were prepared as described by Huynh et al. (2009) with slight modifications. The gel slices were destained with 250 μl each of 40 mM ammonium bicarbonate and 40% acetonitrile (ACN) and dehydrated using 500 μl of 100% ACN for 15 min. The samples were then reduced using 5 mM dithiothreitol (DTT) in 40 mM ammonium bicarbonate buffer at 55°C for 45 min followed by alkylation using 20 mM iodoacetamide (IAA) in 40 mM ammonium bicarbonate buffer under incubation in the dark for 10 min. The supernatant was again discarded followed by addition of 100% ACN and incubation for 15 min. After dehydration, proteins were digested using trypsin (12.5 ng/μl in 50 mM Ammonium bicarbonate buffer) at 37°C/overnight. The digestion was stopped by adding 5% formic acid and the supernatant was collected in a new tube and incubated with 100 μl extraction buffer (5% formic acid and 40% ACN) for 10 min. Final peptide extraction was done by incubating supernatant with 100% ACN for 10 min. The extracted peptides were then purified by ZipTip C18 (Millipore, United States) and the samples vacuum dried and reconstituted in formic acid. After MS analysis, data was collected and analyzed in the NCBI database using Mascot software (Matrix Science).

#### Endotoxin Detection Assay

The presence of endotoxin in the extracted surface proteins from MTCC 5690, MTCC 5689, and NCFM was estimated using Pierce LAL Chromogenic Endotoxin Quantitation Kit (Thermo Fisher Scientific) following the manufacturer’s protocol.

#### Trypan Blue Assay for Testing the Viability of HT-29 Cells With Surface Proteins

The test was performed to check the toxic effects of the total surface proteins of probiotic strains against the human epithelial HT-29 cell lines as described by Strober (1997). At full confluence, the cell line was challenged with surface proteins from each of the three strains at three different concentrations, i.e., 150, 300, and 450 μg/ml PBS and incubated at 37°C/2 h. In order to check the viability of cells, an aliquot of cell suspension was mixed gently with equal volume of 0.2% Trypan blue dye (0.5% in PBS) and kept at room temperature for 3 min. The live and dead cells were counted with the help of a hemocytometer under an inverted microscope. Live cells appeared colorless whereas dead cells appeared blue.

#### *In vivo* Study Using Colitis Mouse Models

##### Experimental animals and induction of colitis (DSS and TNBS mouse model)

Swiss albino mice (6–8 weeks old, male) were procured from the small animal house of ICAR-National Dairy Research Institute, Karnal, Haryana, India after prior approval from the Institutional Animal Ethics Committee (IAEC No. 41-IAEC-18-76). The mice were maintained in accordance with the National Institute of Nutrition, India guidelines for the care and use of laboratory animals. The animals were housed in polypropylene cages and fed normal diet and water *ad libitum*. The animals were grouped into five groups (eight mice each), i.e., colitis group, non-colitis control group (PBS fed), and the three test groups each administered with surface proteins of MTCC 5690, MTCC 5689, and NCFM, respectively. The three treatment groups were oro-gastrically fed with the surface proteins (300 μg/100 μl PBS or 12 mg/kg mice body weight) of respective probiotic cultures for 7 days alternatively before the start of DSS administration, while the colitis control group received PBS during the same period (Supplementary Figure 1A). After 7 days of pre-treatment with surface proteins, the treatment groups were provided with 5% DSS dissolved in drinking water for standard 8 consecutive days until the symptoms were apparent in the colitis control group. Thereafter, DSS was withdrawn and surface proteins were again administered alternatively for another 7 days. The non-colitis control group received PBS oro-gastrically throughout the experimental period. In case of the TNBS model, non-colitis and colitis control groups of mice were orally intubated with PBS alone for the experimental period of 14 days. The three treatment groups were administered at the dose of 300 μg/100 μl/mice or 12 mg/kg mice body weight with respective surface proteins from probiotic strains by oral gavage alternatively for 14 days (Supplementary Figure 1B). On the 15th day, mice were anesthetized and colitis was induced using TNBS (150 mg/kg of body weight, Sigma, United States) dissolved in 50% ethanol through intrarectal administration using a 3.5 F catheter inserted 4 cm proximal to the anus. The non-colitis control group mice were intrarectally administered with 50% ethanol. The severity of colitis in each mouse in both the models was assessed daily using disease activity index (DAI) (Pradhan et al., 2019b), which is the aggregate of the mice body weight loss, stool consistency, and blood in stool on a scale of 0–4 (Supplementary Table 1). In addition, other disease symptoms such as general activity, appetite, and external appearance of mice were also monitored to check the disease progression. The animals were humanely sacrificed on the 23rd day by ether overdosing in DSS model and after 72 h of colitis induction in TNBS model. The mice were then carefully opened by a ventral midline incision using sterile forceps and scissors. The colon, from the colo-cecal junction to the anus, was excised and its length measured after which the colonic contents were flushed out with sterile PBS (1×, pH 7.4). A small part, 2–3 cm, of the colon (rectal region) was kept in 10% buffered formalin for histopathological examination and the remaining tissue was cut into two parts (proximal and middle section) and immediately wrapped in aluminum foil and snap frozen in liquid nitrogen for ELISA testing and myeloperoxidase activity.

##### Histopathology evaluation

The colonic tissues excised from each test animal were fixed in 10% neutral buffered formalin followed by cassetting, mounting, and staining with hematoxylin and eosin. The stained tissue was microscopically examined and scored by a pathologist to study the manifestation of the disease, recognition of various tissue types, the morphologic changes, extent of colonic damage, and inflammation (Maerveld et al., 1999).

##### Myeloperoxidase (MPO) enzyme activity

The MPO activity in the homogenized colonic tissue was checked by suspending it in 0.5% HTAB buffer and determined spectrophotometrically at 450 nm. Briefly, the colonic tissues (proximal region) were retrieved from liquid nitrogen and allowed to gradually thaw. The tissue was then homogenized in nine volumes of ice-cold, 50 mM potassium phosphate buffer (pH 6.0) containing 0.5% hexadecyltrimethyl ammonium bromide. It was then sonicated in an ice bath for 10 s, followed by the freeze-thawing cycle three times and then sonicated again for 10 s. The suspension so obtained was centrifuged at 40,000 × *g*/15 min and the supernatant was collected and analyzed for MPO. The tissue homogenate and all the reagents were brought to 25°C before performing the MPO assay. An aliquot of 7 μl of tissue homogenate was added into a 96-well plate. It was then mixed with 200 μl of O-dianisidine dihydrochloride reaction mixture containing H_2_O_2_. The absorbance was taken at 450 nm using a spectrophotometer. Three consecutive readings were taken at an interval of 30 s. MPO activity was measured in units (U) of MPO/mg tissue, where one unit of MPO was defined as the amount needed to degrade 1 μmol of H_2_O_2_ per min at room temperature considering that 1 unit of MPO = 1 μmol of H_2_O_2_ split and that 1 μmol of H_2_O_2_ gives a change of absorbance of 1.13 × 10^–2^ nm/min. Units of MPO in each sample was determined as change in absorbance [ΔA450 ÷ 0.5 ÷ 0.0113÷ 0.05] (Pradhan et al., 2019a).

##### Estimation of inflammatory markers in colonic homogenate by ELISA (enzyme linked immunosorbent assay)

The concentration levels of the pro-inflammatory (TNF-α) and anti-inflammatory (IL-10) cytokines in the colonic homogenates of non-colitis, colitis, as well as the three treatment mice groups was determined by sandwich ELISA. The colon tissues (middle region) of experimental animals were collected from liquid nitrogen storage and allowed to thaw. The thawed tissues were gently teased with sterile needles and the contents were centrifuged at 2,000 × *g* for 30 min. The supernatant, i.e., colonic extract, was collected and analyzed for the aforementioned cytokines using the commercial kit (BioLegend’s ELISA MAX^TM^ Deluxe Set) following the manufacturer’s protocol.

### Statistical Analysis

All experiments were carried out in triplicate and the results obtained from this study have been presented as mean ± standard deviation (SD). The statistical differences between mice groups were evaluated by one-way ANOVA using Prism 5.0 software (GraphPad Software) following Duncan’s multiple range test. The level of significance was set *a priori* at *P* < 0.05.

## Results

### Extraction, Quantification, and Profiling of Surface Proteins From Probiotic Strains

**FIGURE 1:**
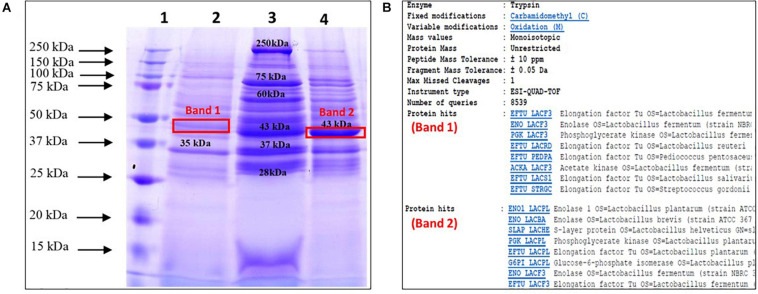
Profiling, identification, and endotoxin determination of surface proteins from test probiotic strains; **(A)** SDS-PAGE profile of surface proteins extracted using LiCl method (Lane 1, Ladder; 2, *L. plantarum* MTCC 5690; 3, *L. acidophilus* NCFM; 4, *L. fermentum* MTCC 5698); **(B)** Identification of selected protein bands using mass spectrometry.

The total surface proteins were extracted from three probiotic *Lactobacillus* strains by 5 M LiCl. Subsequent profiling of the extracted proteins from the three strains on SDS-PAGE yielded numerous protein bands that ranged from ∼25 to ∼250 kDa (Figure 1A). Prominent bands of 35 and 43 kDa were visible in the case of MTCC 5690 and MTCC 5689, respectively, whereas NCFM showed five major bands of sizes 28, 37, 43, 60, 75, and 250 kDa. The protein yield (mg/100 mL culture) was highest for MTCC 5689 (184.6) followed by *L. acidophilus* NCFM (103.05) and MTCC 5690 (91.6). Furthermore, the bands with apparent molecular mass of 45–50 kDa from MTCC 5689 and MTCC 5690 test strains were identified using mass spectrometry as Elongation factor (EF-Tu) (matched with EFTU_LACF3 protein, NCBI Accession No. B2GBC2) and Enolase 1 (matched with ENO1_LACPL, NCBI Accession No. Q88YH3), respectively, with molecular weight of 43.447 kDa. No selected band was identified as SLP with very high score in NCBI/UniProt protein database (Figure 1B), suggesting that test lactobacilli do not possess SLPs on their surfaces, although the SLPs (∼45 kDa) was clearly visible in the NCFM strain.

### Endotoxin Detection and Toxicity of Surface Proteins in HT-29 Cells

The extracted surface proteins were then evaluated for the presence of endotoxins and also checked for their toxicity in HT-29 cells before commencing for *in vivo* experiments. The levels of endotoxin detected ranged from 0.05 to 0.08 EU/mL in different test doses (150, 300, and 450 μg/ml) of proteins (Supplementary Figure 2). The endotoxin levels detected in all the tested concentrations of surface proteins from the three probiotic lactobacilli were in compliance with the FDA’s maximum permissible endotoxin limit of 0.5 EU/mL laid down for any drug product with a maximum human dose of 10 ml/kg^1^. Further, trypan blue exclusion assay was used to determine the cytotoxic effects of extracted surface proteins on the cell viability of HT-29 cell lines. The HT-29 cells when challenged with the aforementioned doses of surface proteins from each of the three strains did not show any negative effects and the cell viability was equivalent to the control without any treatment (data not shown). This suggested the absence of any toxic components or endotoxins in the isolated surface proteins from the *Lactobacillus* strains.

### *In vivo* Efficacy of Probiotic Surface Proteins in DSS and TNBS Colitis Mice Models

A dual model of DSS and TNBS induced colitis mice was used to study the anti-inflammatory effects of total surface proteins from the three probiotic lactobacilli strains. During the course of both the experimental studies, the observations with respect to general health status, colon length, colon histopathology, MPO activity, and cytokines (IL-10 and TNF-α) levels were recorded and compared.

### DSS and TNBS Treated Groups Yielded Non-conclusive Variations in General Health Status

**FIGURE 2:**
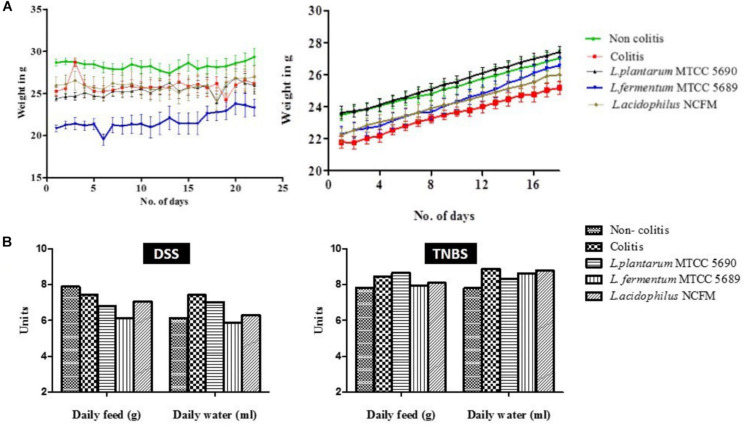
**(A)** Changes in the body weight of mice throughout the experimental study in both DSS and TNBS induced colitis models; **(B)** Average feed and water intake of mice from different groups in both DSS and TNBS induced colitis models.

As a part of the general health status, the gain in mice body weight, feed and water intake, movement and behavior, fur quality, consistency of fecal matter, and presence of blood in stool of mice were monitored. In the DSS colitis model, the DSS administration in the experimental mice did not induce any prominent external colitis symptoms like rectal bleeding or gross diarrhea, rather loose fecal matter along with specks of mucus and sliminess were visible which was more prominent in the colitis group than the rest (DAI of 0, 1.17, 0.73, 0.91, and 0.33 in non-colitis, colitis, MTCC5690, MTCC5689, and NCFM mice group, respectively). In addition, a significant deterioration in the fur quality was noticed in the dorsal side near the tail portion in all the DSS treated groups (maximum in colitis control group and minimum in NCFM treated group) (Supplementary Figure 3). A noticeable decrease in the movement of mice and activity, i.e., slow mobility and docile nature was also observed in colitis control and MTCC 5689 treated groups. Further, inflammation linked tumors in the form of external polyps (hard, irregularly shaped and black in color) were detected in colitis control, MTCC 5689 and MTCC 5690 groups (Supplementary Figure 3). The NCFM treated mice group was observed to be the healthiest with notable hair luster and activity among all the groups. In terms of body weight gain, there was a slight decline in the mice body weight during DSS administration particularly in the DSS group (not significant statistically, *P* > 0.05), however, after the DSS withdrawal, weight increased steadily in all the mice groups (Figure 2A). Overall, no significant conclusions could be drawn from the change in the mice body weight. In case of TNBS induced colitis mice study, no significant differences in the body weight gain was observed between the different treated mice groups throughout the study period (Figure 2A). However, there was steady loosening of stools in day 1 of TNBS administration that resulted in diarrhea in the day 2 along with visible presence of blood in stools that signified the induction of colitis. The colitis symptoms were most prominent in the colitis control (DAI-2.33) and MTCC 5689 (DAI-2.04) mice groups but milder in MTCC 5690 (DAI-1.53) and NCFM (DAI-0.87) treated mice groups. In terms of feed and water intake, no significant variations (*P* > 0.05) were observed between the different treatment groups in both DSS and TNBS model (Figure 2B).

### Changes in the Colon Length of DSS and TNBS Colitis Models

**FIGURE 3:**
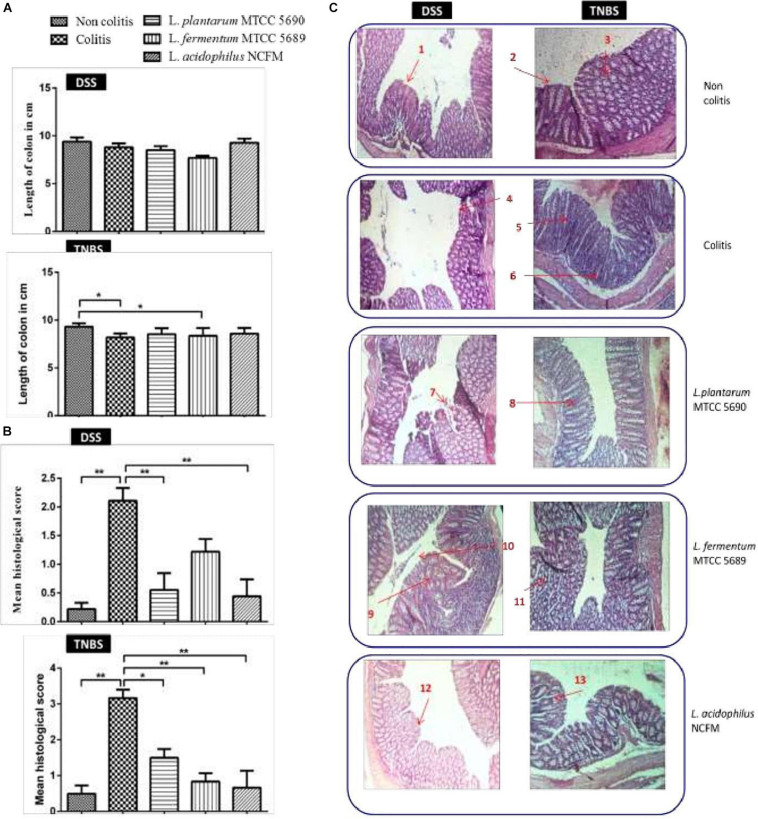
**(A)** Changes in mean colon length of the different mice groups; **(B)** Histopathology-based colitis scores of different mice groups; **(C)** Gross morphology of the colons of mice from different groups used to measure severity of disease (*n* = 8); 1, Intact surface epithelium; 2, Intact crypts; 3, Preserved goblet cells; 4, Erosion of surface epithelium; 5, Infiltration of lamina propria; 6, Crypt abscess; 7, Mild erosion of surface epithelium; 8, Mild loss of goblet cells; 9, Crypt abscess; 10, Mild erosion of surface epithelium; 11, Moderate loss of goblet cells with shortened crypts; 12, Normal colonic mucosa and surface epithelium; 13, Crypt branching with mild loss of goblet cells. *P < 0.05; **P < 0.01.

At the end of the study period, the mice groups were sacrificed by ventral midline incision and observed for macroscopic changes in the colon. The colon from at least six mice each from both the DSS and TNBS colitis control groups showed significant edema with fluid accumulation and evident distension (Supplementary Figure 4), however, no such changes were visible in the colons of other treated mice groups. The colon length observed between the different mice groups (DSS/TNBS) was – non-colitis (9.400 ± 1.202/9.300 ± 0.36 cm), colitis (8.788 ± 1.175/8.186 ± 0.41 cm), and treated groups–MTCC 5689 (7.688 ± 0.6512/8.514 ± 0.63), MTCC 5690 (8.513 ± 1.166/8.357 ± 0.81 cm), and NCFM (9.275 ± 1.208/8.586 ± 0.59 cm). Although, in DSS model, no significant change in either the colitis control or the three treatment groups was observed compared to the non-colitis control, in TNBS model, the colon length of both the colitis and MTCC5689 group was reduced significantly (*P* < 0.05) as compared to the non-colitis control group (Figure 3A). At the same time the MTCC5690 and NCFM group did not show any such reduction in colon length.

### Histological Examination of Impact of Probiotic Surface Proteins in Reversing DSS and TNBS Induced Colitis

The histopathology-based colitis score and evaluation of gross mucosal damage of the colon have been depicted in Figures 3B,C. A blind histopathological examination of the Hematoxylin and Eosin stained colonic tissue from the various mice groups was also carried out by a histologist, who rated the tissues based on the grade of the disease (crypt intactness), 0–4, the percentage of the damage, 0–4, and the severity of the inflammation, 0–3 (Maerveld et al., 1999). In both the studies, the colitogen (DSS and TNBS) treatment had significantly increased the histological score compared to the non-colitis controls. The histological score assessed for DSS model was highest for the colitis group (2.1) followed by MTCC 5689 treated group (1.22) and MTCC 5690 group (0.55). Similarly in TNBS model, the colitis group received the highest score (3.165), followed by MTCC 5690 group (1.50). The maximum reduction in score was exhibited by NCFM treatment, which received scores of 0.44 and 0.665 in DSS and TNBS model, respectively. Although, MTCC 5690 and MTCC 5689 also exhibited significant reduction (*P* < 0.05) in the histological score in both DSS and TNBS model, however, it was not very significant as compared to the NCFM treated group.

The non-colitis control group in both the models revealed normal architecture of colonic epithelium with intact villi, crypts and glands with no evidence of crypt abscess, cryptitis, pseudopolyp, or ulceration. The lamina propria also seemed to be normal with evenly spaced crypts and no notable thickening or thinning was observed. On the contrary, DSS treated colitis group showed ulcerated epithelium with focal lymphoid follicle formation, short and stunted villi, altered mucosa structure and thickened lamina propria. Similarly, TNBS colitis control group also demonstrated significant morphological changes in the colon histology such as erosion of surface epithelium along with ulceration, lumen of crypts infiltrated with neutrophils, dilated and distorted crypts, and loss of goblet cells. There was also an increase in number of inflammatory cells in lamina propria.

#### *Lactiplantibacillus plantarum* MTCC 5690 Treatment

As far as the epithelial changes and the mucosal architecture integrity in MTCC 5690 treated DSS colitis mice was concerned, the biopsy report showed normal colonic epithelium with intact crypts and villi. No evident of crypt abscess or loss of epithelial architecture was noted, however, mild erosion of surface epithelium was visible. Overall improvement and normalization of the epithelial structure was observed. In MTCC 5690 treated TNBS colitis mice, loss of goblet cells along with mild erosion of surface epithelium and comparatively milder infiltration of lamina propria was observed.

#### *Limosilactobacillus fermentum* MTCC 5689 Treatment

In MTCC 5689 treated DSS colitis mice, loss of crypts along with erosion and denudation of surface epithelium, and thin lamina propria was observed. Stricture formation, slight focal inflammatory cell infiltrate with crypt branching and mucosal ulceration was also detected in certain colonic sections. Overall, normalization of epithelium was observed when compared to colitis control group. In MTCC 5689 treated TNBS colitis mice, mild erosion of surface epithelium along with mild loss of goblet cells was observed. However, crypts were seen intact with no apparent ulceration and pseudopolyp formation.

#### *L. acidophilus* NCFM Treatment

In the NCFM treated DSS group, the mice exhibited healthy and intact surface epithelium, normal villi height, intact crypts with no abscess and no evidence of inflammatory infiltration or ulceration. Similarly, in the NCFM treated TNBS group, intact surface epithelium was observed with mild crypt damage and branching at some places along with mild loss of goblet cells. Overall, the NCFM treatment demonstrated significant improvement in the histopathological damages in the colonic tissue in the both the DSS and TNBS models.

### Surface Proteins Effectively Controlled Colitis Mediated Increase in MPO Activity

**FIGURE 4:**
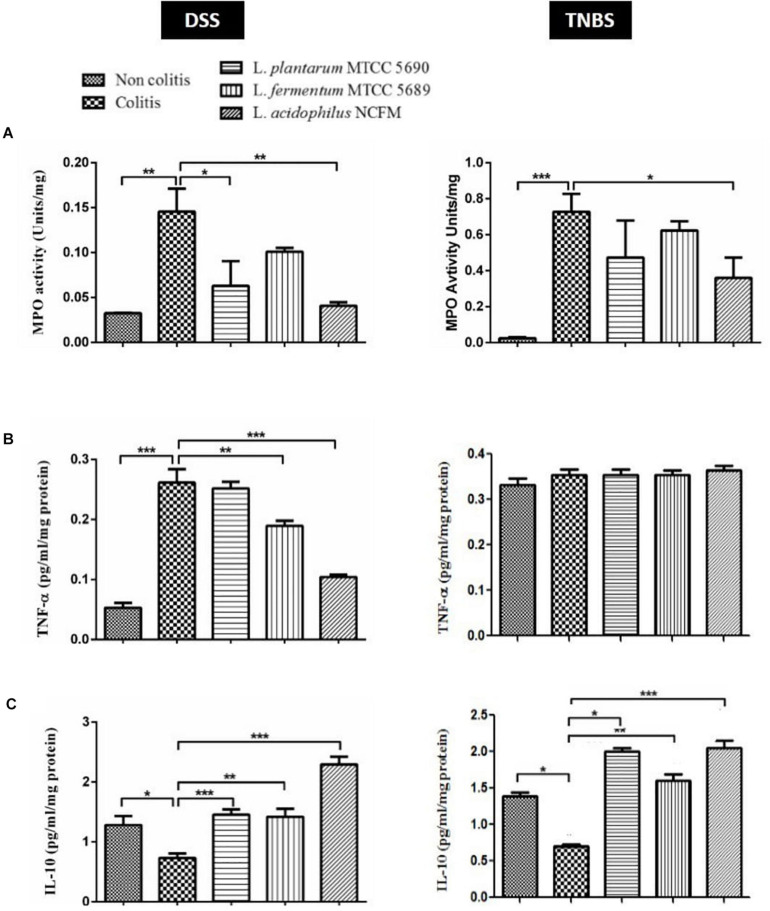
Comparative evaluation of immunomodulatory effects of surface proteins from test probiotic strains in reducing DSS and TNBS induced colitis; **(A)** MPO Activity; **(B)** TNF-α estimation; **(C)** IL-10 estimation *P < 0.05; **P < 0.01; ***P < 0.001.

The MPO enzyme activity was checked in the colonic homogenate spectrophotometrically (Rodriguez-Palacios et al., 2015). The analysis revealed considerable alterations in the MPO activity levels between the control group and the treatment groups in both DSS and TNBS models (Figure 4A). The MPO activity was significantly increased in the DSS colitis group (0.1456 ± 0.05091 U/mg of tissue) compared to non-colitis (0.03246 ± 0.0009483 U/mg of tissue) and other surface protein treated groups *viz.* MTCC 5690 (0.06290 ± 0.05503 U/mg of tissue), MTCC 5689 (0.1009 ± 0.008527 U/mg of tissue) and NCFM (0.04074 ± 0.008087 U/mg of tissue) mice groups. In comparison to DSS colitis group, the remarkable reduction in MPO levels was statistically significant in mice groups treated with surface proteins of NCFM (*P* < 0.01) and MTCC 5690 (*P* < 0.05), although not in MTCC 5689 group (*P* > 0.05).

As anticipated, the intra-rectal TNBS treatment in mice also remarkably enhanced MPO activity, which was observed to be higher in the colitis group (0.7267 ± 0.1002 U/mg) compared to non-colitis control (0.02473 ± 0.006562 U/mg) and other probiotic treated groups *viz.* MTCC 5690 (0.4733 ± 0.2053 U/mg), MTCC 5689 (0.6233 ± 0.05132 U/mg), and NCFM (0.3600 ± 0.1127 U/mg). Overall, the surface proteins from NCFM exhibited most effective reduction (*P* < 0.05) in MPO activity among all the treatment groups. The treatment groups of MTCC 5690 and MTCC 5689 also showed reduction when compared against the colitis control group, however, it was not statistically significant (*P* > 0.05).

### Surface Proteins Modulate the Expression of TNF-α and IL-10 in Colitis Mice Models

The comparative evaluation of cytokines (TNF-α and IL-10) in the colonic homogenate of different mice groups in DSS and TNBS induced colitis model has been illustrated in Figures 4B,C. In DSS colitis model, the levels of pro-inflammatory cytokine, TNF-α was tremendously (*P* < 0.001) increased in the colitis group (0.261 ± 0.06 pg/ml/mg protein) against the non-colitis group (0.052 ± 0.01 pg/ml/mg protein), strongly indicating DSS mediated inflammatory response. Further, significant (*P* < 0.001) suppression in the TNF-α secretion was observed upon the administration of surface proteins from NCFM (0.104 ± 0.01 pg/ml/mg protein), followed by *L. fermentum* MTCC 5689 (0.189 ± 0.02 pg/ml/mg protein). However, the administration of surface proteins from MTCC 5690 did not result in any significant decrease in TNF-α level (0.251 ± 0.03 pg/ml/mg protein). Contrary to this, in TNBS colitis study model, the levels of TNF-α in all the controls and treatment groups did not show any significant differences or alterations.

In case of anti-inflammatory cytokine, IL-10, the levels were significantly (*P* < 0.05) low in the colonic homogenate of DSS colitis mice group (0.731 ± 0.20 pg/ml/mg protein) compared to the non-colitis group (1.28 ± 0.30 pg/ml/mg protein). Further, the oral administration of surface proteins from NCFM (2.29 ± 0.35 pg/ml/mg protein), MTCC 5690 (1.45 ± 0.25 pg/ml/mg protein), and MTCC 5689 (1.41 ± 0.32 pg/ml/mg protein) remarkably (*P* < 0.001) increased the levels of IL-10 than the DSS colitis control group. Similarly, TNBS administration also notably (*P* < 0.001) decreased the IL-10 levels in colitis control group (0.7032 ± 0.05 pg/ml/mg protein) compared to the non-colitis group (1.386 ± 0.16 pg/ml/mg protein). The administration of surface proteins from NCFM (2.050 ± 0.09), MTCC 5690 (1.998 ± 0.05), and MTCC 5689 (1.606 ± 0.08) had a positive impact and tremendously (*p* < 0.001) increased the secretion of IL-10 levels. It is unambiguously clear that surface proteins from *L. acidophilus* NCFM have stronger modulatory effect on the secretion of inflammatory cytokines in the intestine of mice, although, surface proteins from MTCC 5690 and MTCC 5689 also significantly affected cytokine secretion to a variable extent.

## Discussion

Studies have shown that probiotic strains can modulate the intestinal microflora and play a beneficial role in inflammatory conditions such as ulcerative colitis (UC). Although, lactic acid bacteria (LAB) species are considered to be safe, certain threats exists with the use of live probiotic strains such as the risks of opportunistic infections, bacteremia, dissemination of antibiotic resistant determinants, etc (Pradhan et al., 2020), along with challenges of maintaining sufficient viability throughout processing and shelf life of probiotic dietary supplements or food formulations. These issues have gradually prompted the probiotic researchers to explore components or parts of probiotic bacterial cell, which are now widely known as postbiotics, as a probable alternative to live probiotic bacteria. Ample evidences are available that probiotic strains exhibit majority of their probiotic functionality through their cell surface structures, i.e., microbes associated molecular patterns (MAMPs) that bind to specific pattern recognition receptors (PRRs) in the host cells and trigger signaling cascades that results in protective response in different diseased states such as inflammatory conditions (Piqué et al., 2019). Furthermore, the SLPs of probiotic lactobacilli have been experimentally demonstrated to be stable under gastrointestinal transit (Eslami et al., 2013), which may be due to their small and highly stable tertiary structures (Lebeer et al., 2008). Hence, in the present study, the cell surface proteins from certain probiotic strains were used as MAMPs to test for their ameliorative role during inflammatory conditions of colitis using DSS and TNBS induced colitis mouse models.

The two putative test probiotic strains *viz. Lpb. plantarum* MTCC 5690 and *Lm. fermentum* MTCC 5689 used in this study are well known probiotic strains which have been sufficiently proven to possess anti-inflammatory properties under both *in vitro* and *in vivo* conditions. In addition, *L. acidophilus* NCFM, a well-known probiotic was used as a standard strain for comparison. The surface proteins from the probiotic strains were extracted by LiCl process. LiCl interferes with the non-covalent interactions between surface proteins and bacterial surface and have been shown to be superior and less harsh to cells over other extraction reagents like guanidine hydrochloride and lysozyme (Lightfoot et al., 2015). The LiCl extraction process was able to extract intact surface proteins from the probiotic strains which were visible from the SDS-PAGE profile. It was also evident from the SDS-PAGE profiling and further mass spectrometry study that *L. acidophilus* NCFM possessed SlpA (45 kDa) which was absent in the two test probiotic strains, MTCC 5690 and MTCC 5689 as many *Lactobacillus* strains lack SLP, i.e., *Lactobacillus gasseri* and *Lactobacillus johnsonii* (Boot et al., 1995). EF-Tu and Enolase 1 are both moonlighting proteins that are involved in the adhesion of probiotic lactobacilli when expressed over the cell surface. Since our probiotic strains have been studied previously and possessed excellent adhesion characteristics, our findings here on surface proteins also confirmed again the probiotic potential of the test lactobacilli. Nevertheless, the extraction process was able to extract a sufficient amount of surface proteins from the probiotic strains, although the yield was comparatively higher in MTCC 5689 which indicated that the recovery varies in different strains. The surface proteins are a mix of many proteins which may also carry endotoxins (LPS) as such since many may originate from the cell membrane. The safety assessment of extracted surface proteins showed low presence of endotoxins that was within the endotoxin limit of 0.5 EU/mL which is set for any drug product that has a maximum human dose of 10 ml/kg by FDA. Furthermore, no loss of viability in HT-29 cells against different concentrations of surface proteins from the three test strains suggested that the surface proteins did not possess any endotoxins which demonstrated their non-toxic nature and paved the way for subsequent immunomodulatory studies on DSS and TNBS induced colitis mice models.

Chemically induced IBD models using DSS and TNBS are the two most commonly used models due to their simplicity of inducing immediate inflammation with high reproducibility (Trabelsi et al., 2017). DSS-induced colitis model, as such, is widely used because of its many similarities with human UC (Chassaing et al., 2014), likewise, TNBS-colitis model shares homology to human Crohn’s disease due to enhancement in Th1/Th2 ratio (Antoniou et al., 2016). TNBS induced colitis models have also been frequently used to study probiotic interventions during acute inflammation that is apparent during colitis conditions (Biagioli et al., 2017). During diseased conditions the variations in appetite, activity of the mice, and body weight are important external indicators of health status. However, in our study we could not observe either significant loss in mice body weight or changes in the feed and water intake in both the models. Further, symptoms like blood in feces were also not induced in DSS colitis model. Poor induction of colitis symptoms by DSS administration has also been reported by Laroui et al. (2012), who used 3% DSS in mice for 8 days. Alternatively, the distinct development of other disease related symptoms such as low mobility, decline in fur quality, and loose stools specifically in DSS positive control mice groups in both the colitis models suggested the development of diseased conditions. Colon length shortening is one of the biological symptoms of colitis (Chassaing et al., 2014). Although no significant reduction in the mean colon length was observed in the mice groups of DSS model, in TNBS model, a significant shortening of colon length in colitis control and MTCC5689 group was observed. The absence of colon shortening in MTCC5690 and NCFM treated mice groups (TNBS model) indicated that the surface proteins from these groups were able to revert the TNBS induced colon shortening to a certain extent. Further, the colons, in particular in the colitis positive control mice groups in both the colitis models, were found to be visibly distended with fluid along with visible appearance of blood specks. These symptoms have also been reported in colitis conditions by earlier investigators (Ogawa et al., 2004; Erben et al., 2014) which was apparently attenuated in the three surface proteins treated mice groups. At the microscopic level, the histopathological studies showed significant disparity in the colonic microstructure between the different mice groups in both the colitis models. Typical histological changes such as epithelial erosion, neutrophil infiltration into mucosal epithelium, and crypt abscesses were clearly observable in both the colitis control mice groups, which are consistent with the observations made by earlier reports during colitis conditions (Perše and Cerar, 2012; Shon et al., 2015). During the course of IBD, fibrosis and stricture formation of the bowel is a common complication, leading to partial or complete obstruction of the lumen. The external polyp formation reported in this study in the DSS induced colitis control group and two of the treated groups can also be related to the similar kind of intestinal stricture formation and obstruction, which was also observed by Talapka et al. (2016). Håkansson et al. (2011), has also reported polyp formation in mice because of the increased cryptal cell proliferation, changes in crypt cell metabolism and alterations in bacterial flora that predispose an organ to cancer development and has been indicated as an indicator of malignancy in colitis. On the other hand, the probiotic surface proteins treated mice groups showed significant improvement in the colon microstructure as revealed by its histology. This was also strongly supported by the histological scores of both the DSS and TNBS study groups. The histological scores hinted at a significant increase in colitis symptoms in the colitis positive controls of both the DSS and TNBS models, whereas the application of surface proteins from the probiotic strains seemed to have significantly alleviated the colitis condition, wherein the NCFM group showed the best response. Earlier, SlpA derived from the cell surface of *L. acidophilus* NCFM has been reported to play an exceptional role in prevention of histological damages during acute colitis (Lightfoot et al., 2015). The surface proteins of the two test probiotic strains were found to be devoid of the SLPs, nevertheless other surface proteins could also positively ameliorate the histological damages to a certain extent.

The increase in MPO levels due to increased infiltration of neutrophils is a well-established marker for monitoring the extent of inflammation during colitis condition. Many studies have reported elevated MPO levels during colitis condition (Kim et al., 2012; Barone et al., 2018), wherein probiotic interventions had led to a decrease in the MPO activity that corresponded to a decrease in inflammation. Similarly, in the present study, probiotics derived surface proteins were also able to reduce the MPO levels which was highest for NCFM followed by MTCC 5690 and MTCC 5689. Parkos (2016), previously have shown that the chemokine, IL-8 released during infection binds the neutrophils and directs it to the infection site where the neutrophils release MPO. Additionally, Kim et al. (2017) have showed that certain surface structures of *Lactobacillus* strain affect the IL-8 expression, thereby suppressing the MPO levels. Hence, further investigations of the cytokine levels were conducted to verify the definite routes for the anti-inflammatory action. TNF-α, the most potent pro-inflammatory cytokine, along with IFN-γ is involved in the upregulation of pro-inflammatory mediators (iNOS and COX-2) through NF-κB pathway in UC (Cai et al., 2018). In addition, TNF-α has also been implicated in enhanced neutrophil influx at the sites of inflammation (Griffin et al., 2012). On the other hand, IL-10 is an anti-inflammatory/regulatory interleukin whose optimum expression is considered important for maintaining gut homeostasis and also responsible for the anti-inflammatory effects of many probiotic cultures (Konstantinov et al., 2008). In our study, the TNF-α levels did not show any response during TNBS colitis induction, although, its level was significantly increased in DSS-control mice. Further, in the DSS induced colitis study, the three surface proteins treated mice groups showed a decrease in the TNF-α, which was highly significant in NCFM followed by MTCC5689 mice groups. Likewise, the level of IL-10 was significantly downregulated in the colitis control groups in both models, whereas a significant increase was observed in NCFM surface proteins treated mice group. The test probiotic strains also showed significant increase in IL-10 levels, which was at par with NCFM group particularly in TNBS model. Earlier, SLP from *Lactobacillus crispatus* JCM 2009 (Wang et al., 2021) and *Lactobacillus helveticus* MIMLh5 (Taverniti et al., 2013) has been reported to exert anti-inflammatory response by lowering the TNF-α levels that ultimately decrease the activation of NF-κB pathway. Further, the SlpA from *L. acidophilus* NCFM was shown to lower the inflammatory responses by increasing the IL-10 levels which also acts by blocking the NF-κB activity (Konstantinov et al., 2008). Hence, it can be postulated that the anti-inflammatory responses observed in this study could be attributed to the decrease in NF-κB pathway activation either by a decrease in TNF-α expression or an increase in IL-10 levels. The surface proteins from the strain *L. acidophilus* NCFM exhibited the best anti-inflammatory response among the tested strains which was persistently visible in all the parameters tested in both the models. Previously, Lightfoot et al. (2015) have elaborated the role of SlpA (∼45 kDa protein) as a key effector molecule expressed by *L. acidophilus* NCFM in prevention of colitis in murine model. The study reported the balancing effect of *L. acidophilus* SLP on the circulating cytokines levels of TNF-α and IL-10 in the treated mice groups. Although, it was not experimentally proven in this study, it could be presumed that the effect observed w.r.t. NCFM may have been due to the SLPs, however, this needs further verification. Although, the NCFM strain showed the best response, the test strains also exhibited a fairly well anti-inflammatory action that was evident from the improved levels of parameters such as MPO levels, cytokines and histopathology results. Between the two test strains, the cumulative evidences on the parameters analyzed suggests that the surface proteins derived from MTCC 5689 possessed better anti-inflammatory potential compared to MTCC 5690, which corroborate with one of our earlier study by Chauhan et al., 2014), who elaborated strong anti-oxidative and anti-inflammatory role of *Lm. fermentum* MTCC 5689 in the mice gut during colitis. Further, the results of the study also hints that apart from the SLPs, there may be other effector molecules like Ef-Tu or Enolase in the surface proteins of probiotic lactobacilli that may have the potential to regulate the inflammatory responses, however, these effector molecules need to be validated thoroughly.

## Conclusion

The results of the present study demonstrated that the surface proteins of probiotic lactobacilli strains *viz. Lpb. plantarum* MTCC 5690, *Lm. fermentum* MTCC 5689, and *L. acidophilus* NCFM were effective in the amelioration of colitis symptoms. However, there was varied anti-inflammatory response indicating the differences in surface proteins extracted from different strains tested, since the surface proteins are known to vary among different *Lactobacillus* species. Among the three probiotic strains, the surface proteins from *L. acidophilus* NCFM showed consistent activity in both the models. Nevertheless, the results obtained with the two test probiotic strains in the two models signified that there may be other proteins in the probiotic cell surface that may also exhibit potential anti-inflammatory action. Further research on the identification of specific effector proteins and its specific molecular mechanism of action is necessary so as to design novel strategies that are safer preventive and therapeutic options as compared to live cells of probiotics for combating the inflammatory disorders.

## Data Availability Statement

The datasets used in this study to compare the two protein sequences can be found in online repositories. The names of the repository/repositories and accession number(s) can be found below: NCBI Accession No: B2GBC2 and NCBI Accession No: Q88YH3.

## Ethics Statement

The animal study was reviewed and approved by the Institutional Animal Ethics Committee (IAEC Approval No: 41-IAEC-18-76), Indian Council of Agricultural Research – National Dairy Research Institute, Deemed University, Karnal-132001, Haryana, India (Reg. No.1705/GO/ac/13/CPCSA/2013).

## Author Contributions

PRC, DP, KS, SGu, AG, RC, and GG performed the experiments. PRC, DP, KS, AG, SK, and RHM analyzed the data. DP, KS, RHM, JKK, and SGr designed the experiments. PRC and DP performed the statistical analysis. PRC, DP, SK, and SGr wrote the manuscript. SGr conceived the research. All authors approved the final version of the manuscript.

## Conflict of Interest

The authors declare that the research was conducted in the absence of any commercial or financial relationships that could be construed as a potential conflict of interest.

## Publisher’s Note

All claims expressed in this article are solely those of the authors and do not necessarily represent those of their affiliated organizations, or those of the publisher, the editors and the reviewers. Any product that may be evaluated in this article, or claim that may be made by its manufacturer, is not guaranteed or endorsed by the publisher.

## Footnotes

1
https://www.fda.gov/ICECI/Inspections/InspectionGuides/InspectionsTechnicalGuides/uc072918.html
