# Supplementary material for: Ameliorative Effect of Surface Proteins of Probiotic Lactobacilli in Colitis Mouse Models
Source: Front Microbiol. 2021 Sep 3;12:679773. doi: 10.3389/fmicb.2021.679773 (PMC8447872; doi:10.3389/fmicb.2021.679773)
Supplement: Supplementary file 1 [file Data_Sheet_1.docx]

**SUPPLEMENTARY FILES (FIGURES & TABLES)**

**Supplementary Table 1: Disease activity index (DAI) Score card for assessing severity of colitis**

| **Parameter** | **Change** | **Score** |
| --- | --- | --- |
| Weight loss | No change | 0 |
|  | 1 to <5 % | 1 |
|  | 5 to <10 % | 2 |
|  | 10 to <20 % | 3 |
|  | >20 % | 4 |
| Stool Consistency | Negative | 0 |
|  | Loose | 2 |
|  | Diarrhoea | 4 |
| Blood in stool | Normal | 0 |
|  | Blood traces | 2 |
|  | Gross bleeding | 4 |

**
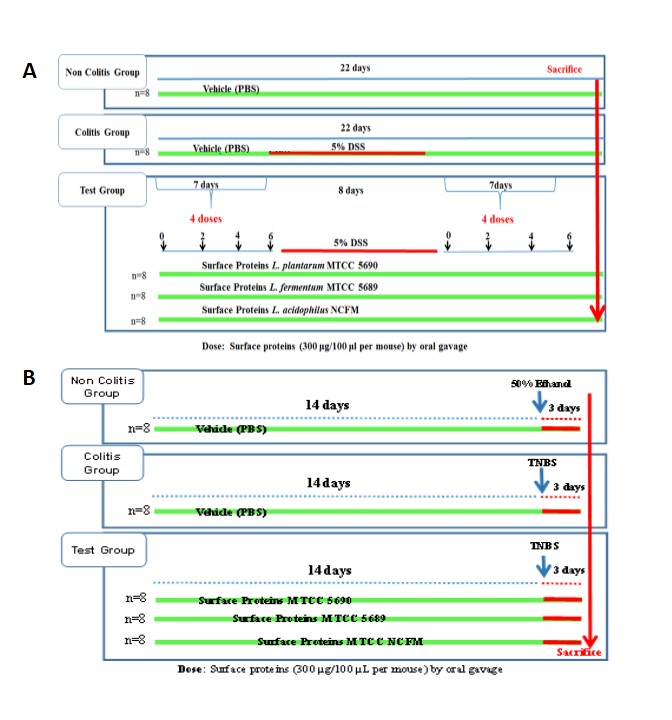
**

**Supplementary Figure 1: Experimental Design for induction of colitis A) DSS mouse model; B) TNBS mouse model**


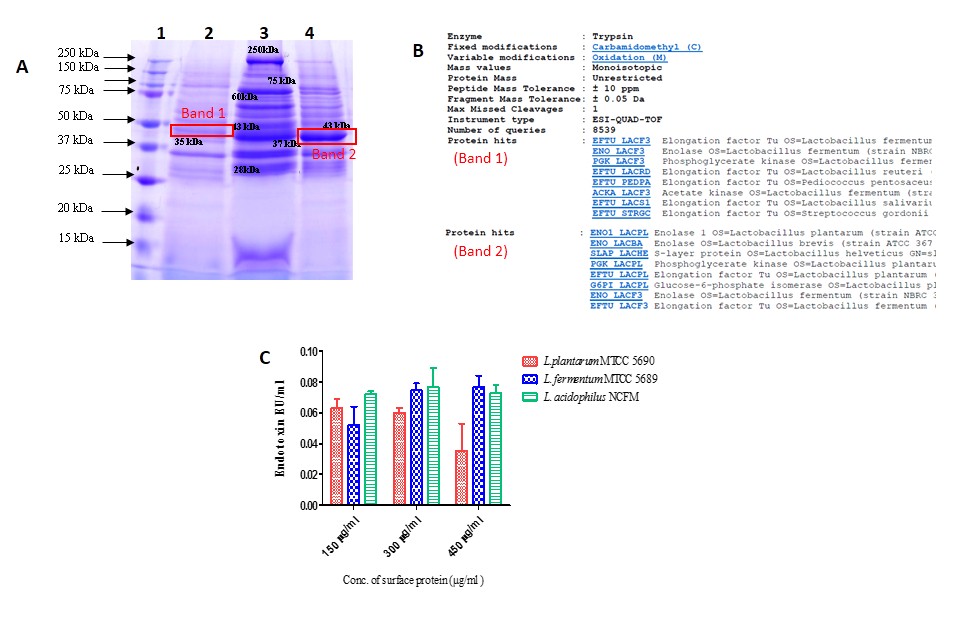


**Supplementary Figure 2: Endotoxin levels present in surface proteins at three different concentration (150, 300 and 450 μg/ml)**

| 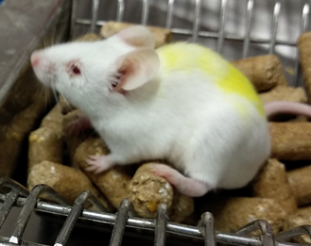  **a** | 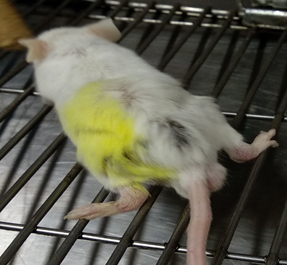  **c**  **b** | | 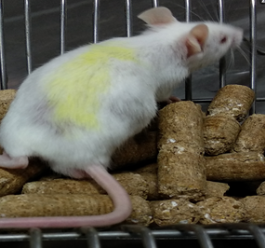 |
| --- | --- | --- | --- |
| 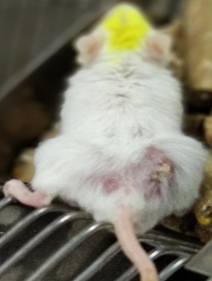  **d** | | 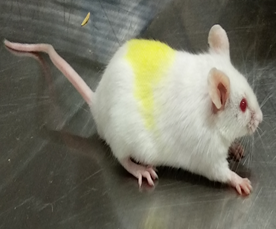  **e**  **e** | |

**Supplementary Figure 3: Change in the fur quality in all group of DSS treated mice; (a) Non colitis group, (b) colitis group, (c) *L. plantarum* MTCC 5690, (d) *L. fermentum* MTCC 5689 and (e) *L. acidophilus* NCFM)**

| 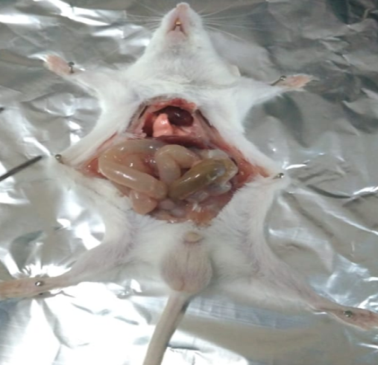 | 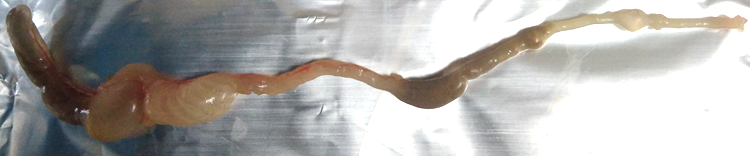 |
| --- | --- |
| **Supplementary Figure 4: Distended Colon in mice groups of Colitis control micegroups** | |
